# Hydroxypropyl Cellulose‐Based Meter‐Long Structurally Colored Fibers for Advanced Fabrics

**DOI:** 10.1002/advs.202404761

**Published:** 2024-10-21

**Authors:** Qinan Qin, Yan Xu

**Affiliations:** ^1^ State Key Laboratory of Inorganic Synthesis and Preparative Chemistry Jilin University Changchun 130012 P. R. China

**Keywords:** hydroxypropyl cellulose, optical patterning, structurally colored fabrics, structurally colored fibers, tunable optical properties

## Abstract

Structurally colored fibers are attractive alternatives to chemically colored fibers due to their rich optical properties, color stability, and environmental friendliness. However, the fabrication of structurally colored fibers using cost‐effective raw materials with the possibility to scale up remains challenging. Here, a simple and scalable approach is developed to fabricate continuous meter‐long structurally colored fibers exhibiting brilliant structural colors across the visible spectrum and helix orientation‐dependent polarization states. The fibers are fabricated by extrusion of concentrated aqueous solutions of chemically crosslinked hydroxypropyl cellulose (HPC). The wavelengths and polarization states can be tuned by solution concentration, relaxation time, and collector's surface energy. The HPC‐based structurally colored fibers display excellent optical stability to mechanical straining, repeated drying/water impregnation, and prolonged heating at 150 °C. It is demonstrated that the HPC‐based structurally colored fibers can be woven into structurally colored fabrics with wavelength‐ and polarization‐coded optical patterns. The current work presents a strategy to tune the chiral nematic order, which constitutes an important step toward mass production of structurally colored fibers with stable and rich optical properties using easily available raw materials.

## Graphical abstract

HPC‐based structurally colored fibers exhibit brilliant structural color and helix orientation‐dependent polarization states. The HPC‐based fibers can be woven into structurally colored fabrics with color‐ and polarization‐coded optical patterns. The strategy for producing continuous structurally colored fibers with tunable optical properties and optical stability constitutes an important step toward the development of structurally colored fabrics for advanced materials.

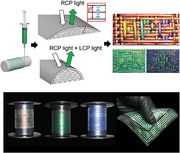

## 1 Introduction

Colored fibers, indispensable raw materials in the textile industry and everyday life have traditionally relied on chemical dyeing processes.^[^ ^1^, ^2^ ^]^ However, these chemically colored fibers pose challenges due to color fading and environmental concerns.^[^ ^3^ ^]^ Structural color, originating from the interactions between visible light and periodic nanostructures, has garnered significant interest due to its distinct advantages including non‐photo‐bleaching, environmental friendliness, and iridescent effect.^[^ ^4^ ^]^ Structurally colored fibers are emerging as a charming alternative, circumventing the issues associated with chemically colored fibers and enriching the functionality of colored fibers for broader applications.^[^ ^5^, ^6^ ^]^

Structurally colored fibers based on photonic crystals are found in many living organisms, such as Saharan silver ants,^[^ ^7^ ^]^ peacock feathers,^[^ ^8^ ^]^ and mallard neck feathers.^[^ ^9^ ^]^ Inspired by nature, structurally colored fibers developed so far are predominantly based on photonic crystals such as colloidal particles^[^ ^6^, ^10^, ^11^ ^]^ and periodic nanolayers.^[^ ^5^, ^12^ ^]^ Chiral nematic liquid crystals, a promising class of photonic crystals, are uniquely positioned due to a combination of helical handedness and 1D photonic bandgaps.^[^ ^13^ ^]^ They enable light modulation in multiple ways, for example, selective reflection of circularly polarized light of the helical handedness, and helix orientation‐dependent reflection of elliptically polarized light.^[^ ^14^ ^]^ Recently, the stimulus‐responsiveness of chiral nematic liquid crystal‐based fibers has been attended to in a handful of research publications.^[^ ^15^, ^16^, ^17^, ^18^ ^]^ However, an understanding of the chiroptical property modulation toward the rational organization of chiral nematic liquid crystal‐based fibers is lacking. Lagerwall and co‐workers designed new chiral nematic liquid crystals, with which mechanically robust structurally colored fibers were produced on a lab scale.^[^ ^15^ ^]^ However, the tedious synthesis procedure and cost involved hinder their large‐scale adoption. To date, a scalable approach using cost‐effective raw materials for the production of structurally colored fibers with tailorable chiroptical properties remains a challenging issue.

Hydroxypropyl cellulose (HPC), a cellulose ether derivative, is a commercially available liquid crystal mesogen with biocompatibility and sustainability‐related advantages.^[^ ^19^ ^]^ In concentrated aqueous solutions, HPC can self‐assemble into a right‐handed chiral nematic phase that enables selective reflection of right‐handed circularly polarized light upon normal incidence.^[^ ^20^, ^21^ ^]^ The wavelength of the selective reflection, *λ*_max_, can be calculated using the Bragg Equation (1):

(1) $$\lambda =n_{\mathrm{avg}}\;\;P\;\;\sin\;\;\theta$$

where *n*_avg_ is the average refractive index, *P* is the helical pitch, *θ* is the angle of incidence from the surface plane,^[^ ^22^ ^]^ and *λ*_max_ occurs at *θ *= 90°. The helical pitch *P* can be tuned by varying the concentration of the HPC solutions.^[^ ^23^ ^]^ The liquid crystalline order can be retained in the solid state through kinetic trapping or covalent crosslinking.^[^ ^24^, ^25^, ^26^, ^27^ ^]^ As an attractive chiral nematic liquid crystal mesogen, HPC has been used to develop films, microspheres, and 3D objects with promising potentials for applications in chiroptical filters, optical sensors, photonic pigments, anti‐counterfeit coatings, and 3D printing.^[^ ^26^, ^28^, ^29^, ^30^, ^31^, ^32^, ^33^, ^34^ ^]^ To the best of our knowledge, HPC‐based continuous meter‐long structurally colored fibers have not yet been reported.

**Figure 1:**
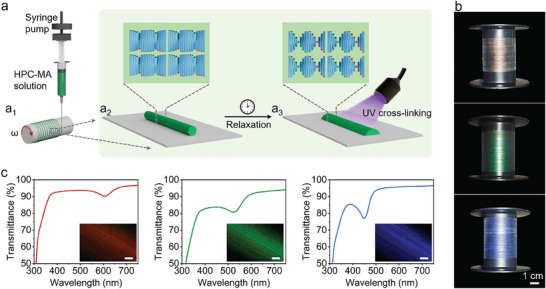
Fabrication of HPC‐based structurally colored fibers. a) Schematic illustration of the fabrication of HPC‐based fibers shows extrusion and winding of HPC‐based fibers a_1_), the morphology and mesostructure of the fibers before relaxation a_2_), and the morphology and mesostructure of the fibers after relaxation and UV crosslinking a_3_). b,c) Photographs of continuous meter‐long red, green, and blue fibers in bobbins of 35 mm in diameter (b), and the corresponding transmission spectra of the fibers (c). The insets in (c) are reflection micrographs. Scale bar: 200 µm.

Herein, we report a facile and scalable approach to fabricate continuous meter‐long HPC‐based structurally colored fibers. Methacrylate‐functionalized HPC (HPC‐MA) solutions are used as precursors and the production of continuous meter‐long fibers is accomplished via a three‐step process involving extrusion, relaxation, and crosslinking (**Figure** **1**a). HPC‐based fibers exhibit vibrant structural colors that can be tuned across the visible spectrum by varying the HPC‐MA solution concentrations. The ordering of the mesophase changes with relaxation time and the collector's surface energy, allowing for structurally colored fibers to be formed with either uniform planar alignment or distorted tilted alignment. The combination of prolonged relaxation and hydrophilic collector warrants the formation of HPC‐based fibers with uniform planar alignment enabling selective reflection of right‐handed circularly polarized (RCP) light. A premature relaxation process or hydrophobic collector enables the formation of HPC‐based fibers with distorted tilted alignment leading to ambidextrous reflection of RCP light and left‐handed circularly polarized (LCP) light. The HPC‐based structurally colored fibers show superb optical stability to mechanical straining (tensile strength up to 46.4 ± 7.2 MPa, toughness up to 1.2 ± 0.4 MJ m^−^^3^), repeated drying/water impregnation and heating (5 h at 150 °C). Finally, we demonstrate that the HPC‐based fibers can be woven into structurally colored fabrics with color‐ and polarization‐coded optical patterns.

## 2 Results and Discussion

### 2.1 Fabrication of HPC‐Based Structurally Colored Fibers

HPC solutions in the concentration range of *ca*. 58–70 wt% display visible colors owing to photonic bandgap‐enabled reflection of the visible light, however, drying causes a blueshift leading to the production of transparent fibers with the photonic bandgaps outside the visible spectrum.^[^ ^35^ ^]^ To overcome this problem, HPC was functionalized with methacrylic anhydride to obtain methacrylated hydroxypropyl cellulose (HPC‐MA) based on the reported protocol (Figure S1, Supporting Information, details in Experimental Section).^[^ ^26^ ^]^ HPC‐MA is capable of self‐assembly forming right‐handed chiral nematic liquid crystalline order while the order can be kinetically arrested in HPC‐MA hydrogels through UV crosslinking.^[^ ^26^ ^]^ The transmission spectra of HPC‐MA hydrogel films recorded before and after drying showed that drying caused a blueshift in the peak wavelengths due to water loss (Figure S2, Supporting Information). Optimized HPC‐MA aqueous solutions with *ca*. 62–68 wt% in concentrations were used as precursors for the fabrication of HPC‐based structurally colored fibers. The rheological properties of the *ca*. 62 wt%, *ca*. 65 wt%, and *ca*. 68 wt% HPC‐MA aqueous solutions were characterized. Flow sweeps in the *ca*. 62 wt%, *ca*. 65 wt%, and *ca*. 68 wt% HPC‐MA aqueous solutions exhibited shear‐thinning behavior with increasing shear rate and a characteristic three‐region flow behavior of lyotropic liquid crystals (Figure S3a, Supporting Information). Oscillatory measurements revealed that the *ca*. 62 wt%, *ca*. 65 wt% and *ca*. 68 wt% HPC‐MA aqueous solutions behaved as viscoelastic liquid (the loss modulus *G″* > the storage modulus *G'*) over the range of oscillatory frequencies and amplitudes measured (Figures S3b,c, Supporting Information). This allowed the HPC precursor solution to be extruded through a nozzle and to flow after extrusion.

Continuous meter‐long HPC‐based fibers with controlled chiroptical properties were fabricated using a homemade extrusion spinning device as schematically illustrated in Figure 1a. HPC‐MA was a viscoelastic polymer, which allowed for continuous pulling of long filaments from the solutions.^[^ ^26^ ^]^ The fibers were extruded by a syringe and wrapped around a glass mandrel as the fiber collector. The mandrel rotated at a speed ω and moved perpendicular to the direction of rotation at a constant speed (Video S1, Supporting Information). As the precursor wetted the glass mandrel, the cross‐sections of the fibers attached to the glass mandrel changed from circular to flat arch within minutes. Notably, all the subsequent characterization and display are based on the convex side of the arch‐shaped fibers. The chiral nematic order formed in the solutions de‐assembled due to shear forces.^[^ ^26^, ^36^ ^]^ Therefore, a certain amount of relaxation time was required for HPC‐MA to re‐assemble into the right‐handed chiral nematic order with desired structural colors. Subsequently, the fibers were photopolymerized using 365 nm light for 2 min to kinetically arrest the chiral nematic order. Drying caused a blueshift in the reflected light wavelengths as evidenced by a change in the structural colors of the HPC‐based fibers (Figure S4, Supporting Information). A similar drying‐caused blueshift of peak wavelengths was observed in the corresponding HPC‐MA hydrogel films. For easy access, the peak wavelengths *λ*_peak_ of the HPC‐MA hydrogel films before and after drying as a function of the HPC‐MA solution concentrations were established (see Figure S2, Supporting Information). The dried fibers were easily stripped off in one piece from the mandrel.

Leveraging the lyotropic liquid crystalline properties of HPC‐MA, the pitches of the HPC‐based structurally colored fibers were readily tunable by changing the HPC‐MA solution concentrations. For example, the colors of the continuous HPC‐based fibers were tuned from red, and green to blue by increasing the HPC‐MA solution concentrations from *ca*. 62 wt%, *ca*. 65 wt% to *ca*. 68 wt%, respectively (Figure 1b). Scanning electron microscope (SEM) imaging analysis showed the formation of periodic layered structures characteristic of chiral nematic order with the pitches in an order of several hundred nanometers, and a decrease in the helical pitches with increasing concentrations (Figure S5, Supporting Information). Strong negative signals on the circular dichroism spectra pointed to the formation of right‐handed chiral nematic structures in the HPC‐based fibers (Figure S6, Supporting Information).

The diameters of the HPC‐based structurally colored fibers were synergistically affected by the extrusion speed, the rotation speed of the mandrel, and the inner diameter of the nozzle outlet of the syringe. Taking the rotation speed as an example, the fiber diameter decreased with an increase in the rotation speed while keeping other conditions constant (Figure S7, Supporting Information). We observed that fibers with larger diameters exhibited slower water evaporation rates at the same relaxation time, causing less significant blueshift in the peak wavelength of the fibers. Meanwhile, the depth of the photonic bandgaps increased with increasing fiber diameters due to stronger interference resulting from the greater number of optical periods (Figure S8, Supporting Information).^[^ ^22^ ^]^ Notably, HPC‐based fibers lose water rapidly due to their large specific surface areas. Therefore, effective control of the water evaporation rate that warrants the production of fibers with reproducible chiroptical properties suitable for large‐scale production poses a real challenge. Toward this goal, we explored the addition of glycerol as a humectant or covered the fibers with a layer of non‐volatile oil to prevent water evaporation, however, neither was satisfactory. In what follows, large‐diameter fibers (≈1000 µm) and high humidity conditions (>95% RH) were used to minimize concentration variations caused by water evaporation.

### 2.2 Optical Properties Versus Relaxation Time

**Figure 2:**
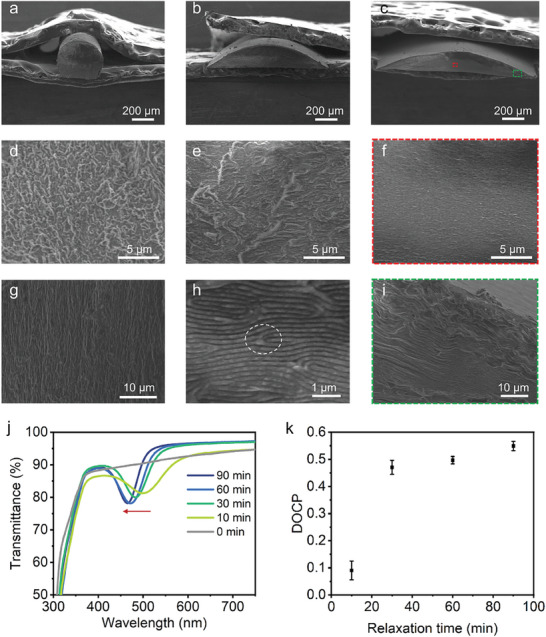
Optical properties of the HPC‐based structurally colored fibers versus relaxation time. a–f) Cross‐sectional SEM images at lower magnification a–c) and higher magnification d–f) of the HPC‐based fibers were produced with the relaxation time of 0, 10, and 90 min, respectively. g) Longitudinal‐sectional SEM image of the HPC‐based fibers produced with the relaxation time of 0 min. h) Cross‐sectional SEM image of the HPC‐based fibers produced with the relaxation time of 10 min, highlighting disclination defects (white dashed circle). i) Cross‐sectional SEM image of the HPC‐based fibers produced with the relaxation time of 90 min (green square in (c)), highlighting wrinkles near the fiber edge. j) Transmission spectra of the HPC‐based fibers produced with the relaxation time between 0 and 90 min. k) A trend in the DOCP value of the fibers as a function of relaxation time.

During the extrusion process, shear forces disrupt the chiral nematic structure of HPC‐MA as observed by Chan and coworkers, necessitating a relaxation time to allow for its reorganization.^[^ ^26^ ^]^ To understand the relaxation time effect on the self‐assembly of HPC‐MA, the mesostructures of HPC‐based fibers during the relaxation process were characterized using SEM (**Figures** **2**a–i). Initially, HPC‐based fibers were cross‐linked in situ immediately after they were attached to the glass mandrel, and colorless fibers were obtained (Figure S9, Supporting Information). SEM images showed cylindrical fibers with nearly circular cross‐sections (Figure 2a). At higher magnifications, disordered mesostructures at the cross‐section (Figure 2d) and stripe patterns at the longitudinal section (Figure 2g) revealed similar to the observations by Chan and coworkers.^[^ ^26^ ^]^ This was attributed to the shear‐induced de‐assembly of chiral nematically ordered HPC‐MA and the formation of parallel alignment along the shear direction during the extrusion process.^[^ ^36^ ^]^ As the relaxation proceeded, the cross‐section of the fibers transformed from circular to flat arch with decreasing curvature and a corresponding change in the mesostructures.

After 10 min relaxation, the HPC‐based fibers exhibited vibrant structural colors (Figure S9, Supporting Information). Periodic layered structure characteristic of chiral nematic order was observed in the central region of the fibers owing to the re‐assembly of HPC‐MA from disordered mesophase to chiral nematic phase.^[^ ^26^ ^]^ The substantial presence of structural defects like disclinations and chiral nematic polydomains was visible (Figures 2e,h). After 60 min relaxation, SEM images showed the formation of chiral nematic monodomain in the central region with its helical axis perpendicular to the surface of the glass mandrel (Figure 2f). Tilted domains and buckling near the fiber edge were observed on the cross‐sectional SEM images of the fibers (Figure 2i). The formation of these structural defects was associated with a higher water evaporation rate at the fiber‐air interface and Helfrich‐Hurault instabilities driven by bulk elastic energy and interfacial effects.^[^ ^37^, ^38^, ^39^ ^]^

Transmission spectra of the HPC‐based fibers were recorded as shown in Figure 2j. Increasing the relaxation time caused a blueshift in the peak wavelength due to water evaporation.^[^ ^23^ ^]^ This was accompanied by a narrowing and deepening of the photonic bandgap due to relaxation‐facilitated growth of the chiral nematic domain, which was consistent with the corresponding cross‐sectional SEM images (see Figures 2d–f). Longer relaxation time facilitated the formation of chiral nematic phases with large domain sizes and fewer defects. Prolonging the relaxation longer than 60 min caused insignificant changes in the chiral nematic order, based on which 60 min was considered optimal for the production of HPC‐based structurally colored fibers.

The HPC‐based fibers formed with planar alignment are capable of selective reflection of RCP light due to their right‐handedness. However, the presence of structural defects and domains of different helix orientations deteriorates the polarization selectivity (Figures S10 and S11, Supporting Information).^[^ ^14^ ^]^ The ability of a chiral nematic phase to selectively reflect circularly polarized light of the helical sense can be assessed based on the degree of circular polarization (DOCP) with Equation (2):

(2) $$\mathrm{DOCP}=\left(I_{R}-I_{L}\right)\;/\;\left(I_{R}+I_{L}\right)$$

where *I*_R_ and *I*_L_ are the peak intensities of RCP light and LCP light by reflection, respectively.^[^ ^27^, ^40^ ^]^ DOCP value is −1 or +1 when white light is normal incidence on a perfect left‐handed chiral nematic phase or a perfect right‐handed chiral nematic phase. Oblique incidence on a perfect planar alignment or normal incidence on a tiled alignment enables ambidextrous reflections of RCP light and LCP light with an angle‐dependent intensity ratio.^[^ ^14^ ^]^

A trend in the DOCP value as a function of relaxation time was established for the HPC‐based fibers. Figure 2k shows that prolonged relaxation produced HPC‐based fibers with higher DOCP values due to the formation of chiral nematic order with large domain sizes and fewer defects in keeping with the corresponding cross‐sectional SEM images (see Figures 2e,f). Excessively long relaxation time led to the production of colorless fibers due to high concentration‐induced blueshift of the photonic bandgaps to the near UV region.^[^ ^35^ ^]^ In addition, prolonged relaxation increased the time cost, making the process less economical. As such, it is important to strike a balance between the two factors and make an informed decision when designing the production parameters to achieve HPC‐based structurally colored fibers with desired optical properties at low cost.

### 2.3 Optical Properties Versus the Surface Energy of Collectors

**Figure 3:**
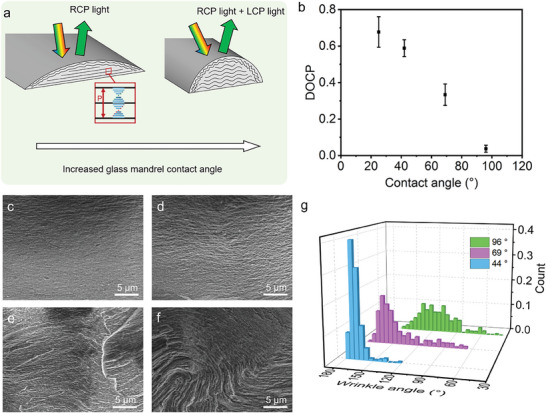
Optical properties of the HPC‐based structurally colored fibers versus the surface energy of the glass mandrel. a) Schematics of the cross‐sectional geometries, mesostructures, and optical properties of the HPC‐based fibers collected using hydrophilic glass mandrel (left) and hydrophobic glass mandrel (right). b) A trend in the DOCP value of the fibers as a function of the contact angles of the glass mandrel surface. c–f) Cross‐sectional SEM images of the HPC‐based fiber collected on the glass mandrel with the contact angles of 29° c), 44° d), 69° e), and 96° f), respectively. g) The wrinkle angle distribution histogram based on the SEM images of HPC‐based fibers collected on the glass mandrel with contact angles of 44°, 69°, and 96°, respectively. The statistics were established based on 250 wrinkles.

The chiroptical properties of the HPC‐based structurally colored fibers changed with the surface energy of the glass mandrel as the collector (**Figure** **3**a). Different degrees of hydrophobic treatments on the glass mandrel surface reduced the surface energy of the glass, resulting in water contact angles of 44°, 69°, and 96°, respectively (Figure S12, Supporting Information). This resulted in fibers with arch‐shaped cross‐sections while the arch curvature increased with increasing hydrophobicity of the glass mandrel surface (Figure S13, Supporting Information). In what follows, the surface energy effects on the chiroptical properties of the fibers were compared at a fixed relaxation time of 60 min.

The reflection microscopy images of the HPC‐based fibers recorded through the 400–700 nm RCP or the 400–700 nm LCP filters (RCP filter, LCP filter) showed that the fibers collected on glass mandrels with different contact angles reflected RCP light and LCP light with different color intensities (Figure S14, Supporting Information). Transmission spectra of the fibers were measured through the RCP filter or the LCP filter, based on which their DOCP values were calculated using Equation (2) (Figure 3b; Figure S15, Supporting Information). The DOCP value decreased with an increase in the surface contact angles of the glass mandrel. It was shown that increasing the hydrophobicity of the glass mandrel increased the proportion of LCP light reflected by the HPC‐based fibers. For example, the HPC‐based fibers collected on the glass mandrel with hydrophilic surfaces (contact angle of 25°) gave a DOCP value of 0.68 ± 0.08, indicating that the fibers predominantly reflected RCP light. In striking contrast, the fibers collected on the glass mandrel with hydrophobic surfaces (contact angle of 96°) gave a DOCP value of 0.04 ± 0.02, indicating that the fibers reflected both RCP light and LCP light with comparable intensities. Hydrophilic glass mandrel promotes the formation of chiral nematic order while hydrophobic glass mandrel leads to the formation of distorted chiral nematic order.

Cross‐sectional SEM images of the HPC‐based fibers collected on the glass mandrel with a contact angle of 25° revealed the formation of uniform planar alignment in the central region of the fibers (Figure 3c), in contrast to the periodic undulation and lamellar buckling in the central region of the fibers formed on glass mandrels with increased hydrophobicity (Figures 3d–f). The distribution of wrinkle angles observed in the cross‐sectional SEM images of HPC‐MA fibers, collected on glass mandrels with different contact angles, was statistically analyzed (Figure 3g). The fibers collected on a hydrophilic substrate (contact angle 25°) exhibited negligible internal wrinkling and were therefore excluded from the statistical range. The results indicated that the wrinkling of the microstructure becomes progressively severe as the surface contact angle increases. This was attributed to hemicylindrical geometries triggering Helfrich‐Hurault instabilities, resulting in frustrated chiral nematic order.^[^ ^27^, ^37^ ^]^ When the helical axis was tilted, reflected light was elliptically polarized consisting of two light waves that are linearly polarized with the same frequency but unequal amplitudes, unlike circularly polarized light.^[^ ^14^ ^]^ Furthermore, distorted helicoids may produce wavelength retardation plates of quarter, half, or other arbitrary phase differences. The combination of the above accounted for the formation of HPC‐based fibers enabling ambidextrous reflection of RCP and LCP light. Similar behaviors have been observed in cellulose photonic systems with constrained geometries.^[^ ^26^, ^35^, ^39^, ^41^ ^]^ Leveraging the light‐matter interactions of chiral nematic phases and wavelength retardation phases presents a strategy to modulate the polarization state of the HPC‐based structurally colored fibers.

### 2.4 Optical Stability of the HPC‐Based Structurally Colored Fibers

**Figure 4:**
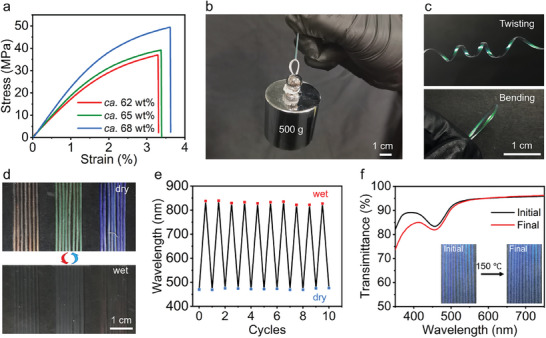
Optical stabilities of the HPC‐based structurally colored fibers. a) Tensile stress‐strain curves of the HPC‐based fibers fabricated from the *ca*. 62 wt%, *ca*. 65 wt% and *ca*. 68 wt% solutions, respectively. b) The photograph shows that a double‐stranded HPC‐based fiber of 1.20 mm in diameter lifts a 500 g weight. c) Twisting and bending of the HPC‐based fibers. d,e) Photographs show the color change with wetting and drying (d) and the cyclic performance of the peak wavelength after 10 wetting‐drying cycles (e). f) Transmission spectra of the fibers before (black line) and after (red line) 5 h heating at 150 °C. Insets are the photographs of the fibers before and after the heating treatments.

Optical stability is an essential parameter used to assess the suitability of structurally colored fibers for practical applications. For easy assessment, the mechanical properties of the HPC‐based fibers fabricated from HPC‐MA solutions of different concentrations were established as summarized in **Figures** **4**a and Table S1 (Supporting Information). Of significance to note, the tensile strength, toughness, and Young's modulus of the fibers increased with the HPC‐MA solution concentrations. For example, fibers from the *ca*. 68 wt% HPC‐MA solution exhibited a tensile strength of 46.4 ± 7.2 MPa, toughness of 1.2 ± 0.4 MJ m^−^^3^, and Young's modulus of 2.1 ± 0.4 GPa; in contrast, fibers from the *ca*. 62 wt% HPC‐MA solution exhibited a tensile strength of 37.0 ± 3.2 MPa, toughness of 0.7 ± 0.2 MJ m^−^^3^, and Young's modulus of 1.8 ± 0.2 GPa. Such an improvement was attributed to the higher cross‐linking density of the hydrogels formed at higher HPC‐MA concentrations.^[^ ^42^ ^]^

The HPC‐based structurally colored fibers exhibited excellent optical stability to mechanical treatments. One illustration showed that a 0.010 g HPC‐based fiber (60 mm in length and 1.20 mm in diameter) was capable of withstanding a 500 g weight (Figure 4b and Video S2, Supporting Information). Their optical appearance and structural integrity remained after repeatedly bending and twisting (Figure 4c). The corresponding cross‐sectional SEM images showed insignificant changes in the chiral nematic order after 50 times the bending and twisting treatments (Figure S16, Supporting Information). It indicated the optical stability of the HPC‐based fibers to mechanical treatments.

The HPC‐based structurally colored fibers exhibited excellent optical stability to repeated drying/water impregnation and heating. In one illustration, the HPC‐based fibers were immersed in water for 2 h at ambient temperatures followed by natural drying, and the process was repeated 10 times. The integrity of the fibers remained intact after the repeated wetting‐drying (Figure 4d). Transmission spectra showed wetting‐induced redshift in the peak wavelength due to water adsorption, which can be reversed completely upon drying (Figure 4e). Furthermore, the HPC‐based fibers maintained their vibrant structural colors after 5 h at 150 °C (Figure 4f). Prolonged heating above 150 °C caused yellowing of the fibers. Heating the fibers at 245 °C resulted in decomposition based on the thermogravimetric analysis (Figure S17, Supporting Information). Indeed, the optical stability to mechanical treatments, wetting, and heating qualified the HPC‐based fibers as a cost‐effective sustainable raw material for the development of structurally colored materials such as fabrics.

### 2.5 HPC‐Based Structurally Colored Fibers for Advanced Fabrics

**Figure 5:**
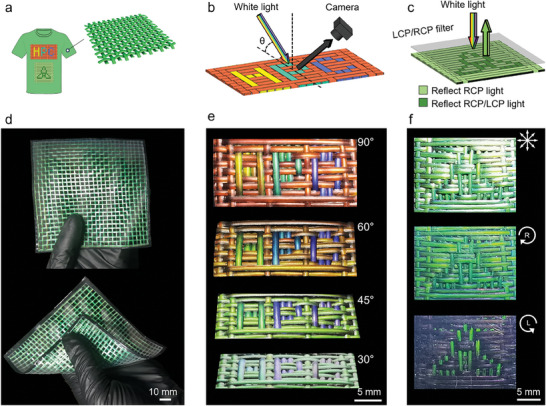
HPC‐based structurally colored fibers for advanced fabrics. a–c) HPC‐based advanced fabrics with color‐ and polarization‐coded optical patterns a), angle‐dependent colors b), and polarization‐coded optical patterns (c). d) Photograph of a woven fabric using single‐colored fibers. e) Photographs of an HPC‐patterned fabric appear differently when viewed at different angles. f) Photographs of a trinity‐patterned fabric when viewed under normal white light (top), and through the RCP filter (middle) or the LCP filter (bottom).

The HPC‐based structurally colored fibers with chirality specificity, multifarious polarization states, and optical stability across the visible spectrum offer unique advantages toward the development of structurally colored fabrics and advanced materials. To demonstrate the application potentials, two sets of HPC‐based fibers in perpendicular orientations were crossed and interwoven to form coherent, stable, and flexible fabrics. Woven fabrics with desired optical patterns and dimensions can be made using fibers of specific colors and polarization states (**Figures** **5**a–c).

As proof‐of‐concept illustrations, a monochromatic fabric of 100 mm x 100 mm in dimension was made by interlacing two sets of green fibers in perpendicular orientations, and the fabric was flexible and foldable (Figure 5d). A fabric with an HPC pattern (26 mm × 12 mm) was created by crossing and interweaving two sets of perpendicular fibers of yellow and red, green and red, and blue and red, respectively (Figures 5b,e). The colors of the HPC‐patterned fabric appeared more intensified or hidden when viewed through the RCP filter or the LCP filter owning to the selective reflection of RCP light (Figure S18, Supporting Information). The HPC‐patterned fabric exhibited angle‐dependent colors, where the color of the fabric blueshifted as the viewing angle changed from 90°, 60°, 45° to 30° with respect to the surface plane of the fabric.

We demonstrated that fabrics with polarization‐coded optical patterns were achievable using the HPC‐based structurally colored fibers. As a proof‐of‐concept, dark green fiber and light green fiber were fabricated from a *ca*. 65 wt% HPC‐MA solution collected on the hydrophilic glass mandrel with a contact angle of 25° and the hydrophobic glass mandrel with a contact angle of 96°, respectively. The two green fibers in perpendicular orientations were crossed and interwoven to form a coherent, stable, and flexible fabric of 21 mm × 1.7 mm in dimension. The dark green fiber that reflected both LCP light and RCP light with a peak wavelength of 525 nm was used for the trinity flower pattern; the light green fiber that reflected RCP light with a peak wavelength of 512 nm was used for the background. The trinity‐patterned fabric appeared differently under different viewing modes (Figures 5c,f). Specifically, the fabric displayed a dark green trinity pattern on a light green background upon normal incidence of white light; when viewed through the RCP filter, the color of the trinity pattern appeared weakened due to a combination of forbidden propagation of LCP light and optical filtration‐reduced light flux; viewing through the LCP filter disguised the fabric to a green trinity pattern on a dark background due to a combination of forbidden propagation of RCP light and optical filtration‐reduced light flux. As a consequence, the trinity‐patterned fabric was transformed into a polarization‐coded pattern that can be precisely read out using a polarimeter.

## 3 Conclusion

A facile and scalable protocol was carefully established to enable the production of continuous meter‐long HPC‐based structurally colored fibers with tunable diameters and optical properties. We showed that the fiber colors arising from light reflection were accessible across the visible spectrum by simply changing the HPC‐MA solution concentrations. More interestingly, the polarization state of reflected light was tailorable to either RCP light or a mixture of RCP/LCP light of varied ratios by controlling the relaxation time and the collector's surface energy. The HPC‐based structurally colored fibers displayed excellent optical stability to mechanical treatments (coiling, bending, and twisting), water impregnation, and heating up to 150 °C. Finally, we demonstrated as a proof‐of‐principle the potential of the HPC‐based structurally colored fibers for structurally colored woven fabrics with color‐ and polarization‐coded optical patterns. In this context, our strategy for producing continuous structurally colored fibers with tunable optical properties and optical stability constitutes an important step toward the development of structurally colored fabrics for advanced materials.

## 4 Experimental Section

### Materials

Hydroxypropyl cellulose (HPC grade: SSL, Mw = 40,000 g mol^−1^) was purchased from Nippon Soda Co., Ltd. Sodium hydroxide and methacrylic anhydride were purchased from Aladdin. 2‐hydroxy‐4′‐(2‐hydroxyethoxy)‐2‐methylpropiophenone (Irgacure 2959) was purchased from Macklin. Commercial water repellent agent Aquapel was purchased from a supplier. Milli‐Q water was used throughout all experiments. All reagents were used as supplied without further purification.

### Synthesis of HPC‐MA

HPC‐MA was synthesized based on the reported protocol.^[^ ^26^ ^]^ HPC was vacuum‐dried overnight. In a typical experiment, 10 g of the vacuum‐dried HPC was dissolved in 500 mL of deionized (DI) water, to which 40 mL of methacrylic anhydride was added. Next, 59 mL of 5 m sodium hydroxide aqueous solution was added dropwise into the solution and stirred overnight at room temperature to allow thorough reaction. The resulting white slurry was dialyzed for five days until the insoluble white matter was eliminated, and it was then lyophilized to obtain loose white flakes of HPC‐MA.

### Preparation of HPC‐MA Solution

HPC‐MA was vacuum dried at 60 °C overnight before use. In a typical experiment, 6.5 g of HPC‐MA was dissolved in 3.5 g of DI water, to which 0.065 g of 1.0 wt% Irgacure 2959 (with reference to HPC‐MA) was added. After extensive mixing, the mixture was centrifuged at 8000 rpm to remove bubbles, and stored at 4 °C in the refrigerator away from light. Then, the HPC‐MA solutions were taken out of the refrigerator and kept at room temperature for 3 h before use.

### Preparation of HPC‐Based Structurally Colored Fibers

To prepare HPC‐based fibers, a simple homemade extrusion spinning device was used. In a typical procedure, the HPC‐MA solution was extruded at a constant speed from a syringe and collected on a glass mandrel controlled by an electric motor. The untreated glass mandrel was hydrophilic (contact angle of 25°), and to obtain the hydrophobic mandrel (contact angle of 96°), the surface of the glass mandrel was treated using the commercial water‐repellent agent Aquapel and incubated for 5 min. By rotating the mandrel at an appropriate constant speed and moving it along the direction perpendicular to the rotation, continuous HPC‐based fibers were wound onto the mandrel. The fibers were allowed to relax for a certain period of time in a high relative humidity environment provided by a constant temperature and humidity chamber (23 °C, 95% RH). Then HPC‐based fibers were crosslinked in situ by the 365 nm light source placed ≈10 cm above the mandrel. The polymerization time was ≈2 min with the power density of 5000 mW cm^−2^. Thus, continuous HPC‐MA structurally colored fibers were obtained. To enhance the saturation of the reflective color and prevent the underlying fiber color from showing through the upper layers, the back (flat side) of the HPC‐based fibers in the woven patterned fabrics shown in Figures 5e,f were colored with black pigment. The other HPC‐based structurally colored fibers mentioned in the paper were not dyed.

### Characterization

UV–vis transmission spectra were measured by analytic Jena specord 210 plus UV–vis spectrophotometer probed by normal light and through the RCP filter and the LCP filter. Circular dichroism spectra were recorded in automatic mode using a BioLogic MOS‐450 spectropolarimeter. Optical micrographs were acquired on Nikon Optiphot 200 Microscopy in reflection mode under normal white light and by inserting the RCP filter and the LCP filter in the light path. Microstructures of the samples were characterized using JEOL‐7800F field emission scanning electron microscopy (SEM) at an accelerating voltage of 3.0 kV. Samples were gold sputtered for 30 s before SEM observation. ^1^H‐NMR spectra were recorded on a Bruker Avance NEO 400 NMR spectrometer. Contact water angle was collected by using a SINDIN SDC‐350 contact angle test system by dropping a 3 µL size of ultrapure water on the surface of the glass. Five positions were tested for each sample. The tensile mechanical properties were tested on a universal material testing machine (SANS, CMT6203, China) equipped with a 500 N load cell. Samples were cut into 20 mm in length. At least five specimens from each sample were tested with a stretching speed of 2.0 mm min^−1^ to obtain reliable data. Thermogravimetric analysis measurement was performed on a TA company TGA Q500 unit in air flow heating from 30 to 800 °C at a heating rate of 10 °C min^−1^.

## Conflict of Interest

The authors declare no conflict of interest.

## Supporting information



Supporting Information

Supplemental Video 1

Supplemental Video 2

## Data Availability

The data that support the findings of this study are available from the corresponding author upon reasonable request.
