# Supplementary material for: Hydroxypropyl Cellulose‐Based Meter‐Long Structurally Colored Fibers for Advanced Fabrics
Source: Adv Sci (Weinh). 2024 Oct 21;11(46):2404761. doi: 10.1002/advs.202404761 (PMC11633506; doi:10.1002/advs.202404761)
Supplement: Supplementary file 1 — Supporting Information [file ADVS-11-2404761-s001.pdf]

ADVANCED SCIENCE
Open Access

Supporting Information

for *Adv. Sci.*, DOI 10.1002/advs.202404761

Hydroxypropyl Cellulose-Based Meter-Long Structurally Colored Fibers for Advanced Fabrics

*Qinan Qin and Yan Xu**

Supporting Information

Hydroxypropyl Cellulose-Based Meter-Long Structurally Colored Fibers for Advanced Fabrics

*Qinan Qin, and Yan Xu**

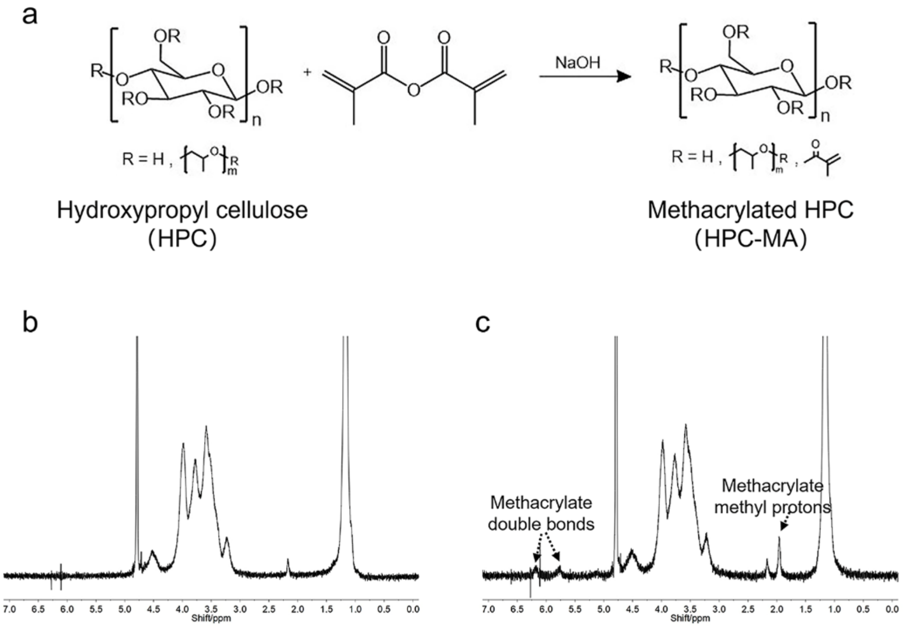

Figure S1. (a) Synthesis process of methacrylated hydroxypropyl cellulose (HPC-MA). (b) ^{1}H-NMR spectra of HPC. (c) ^{1}H-NMR spectra of HPC-MA.

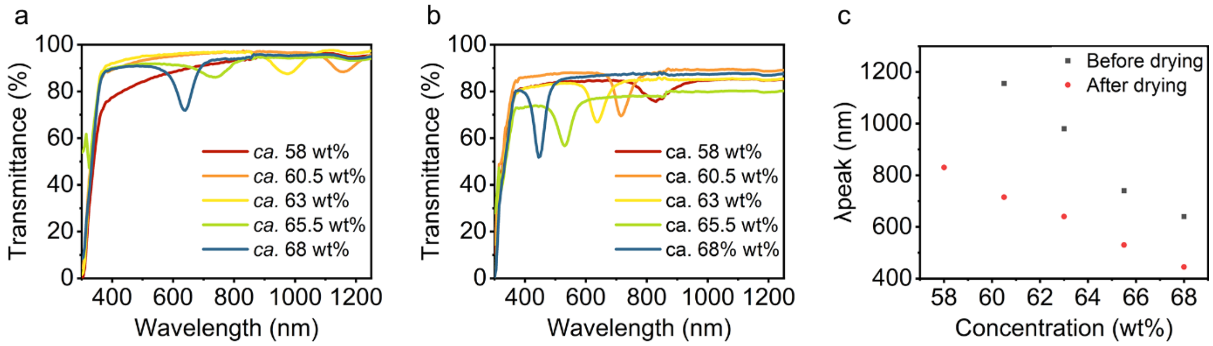

Figure S2. Transmission spectra of HPC-MA hydrogel films before drying (a) and after drying (b) prepared from the HPC-MA solutions of different concentrations. (c) A trend in the peak wavelength λ_{peak} of HPC-MA hydrogel films before (black points) and after drying (red points) as a function of HPC-MA solution concentrations.

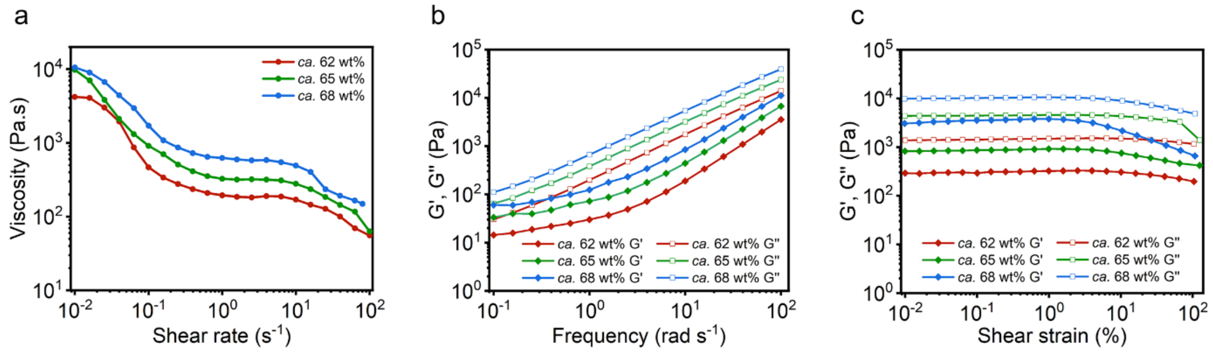

Figure S3. Rheological measurements of the *ca*. 62 wt%, *ca*. 65 wt% and *ca*. 68 wt% HPC-MA aqueous solutions, respectively. (a) Viscosity as a function of shear rate measured through flow sweep experiment. (b, c) Storage modulus (G') and loss modulus (G'') measured over (b) a range of oscillatory frequency at 0.1 % strain and (c) a range of oscillatory amplitudes at a frequency of 10 rad s^{-1}.

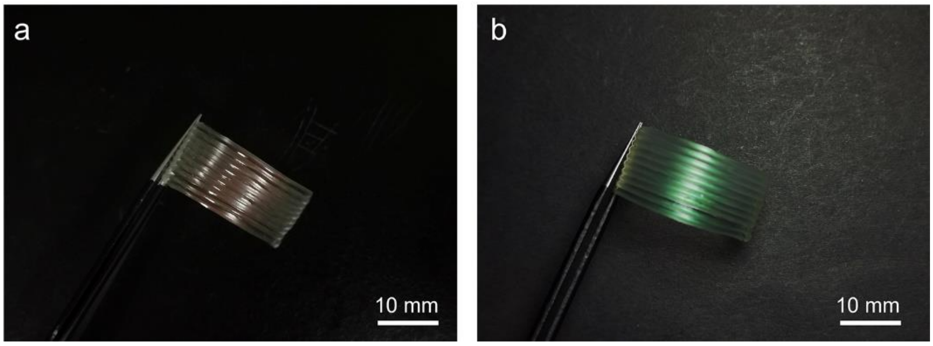

Figure S4. Photographs of the HPC-based fibers before (a) and after drying (b).

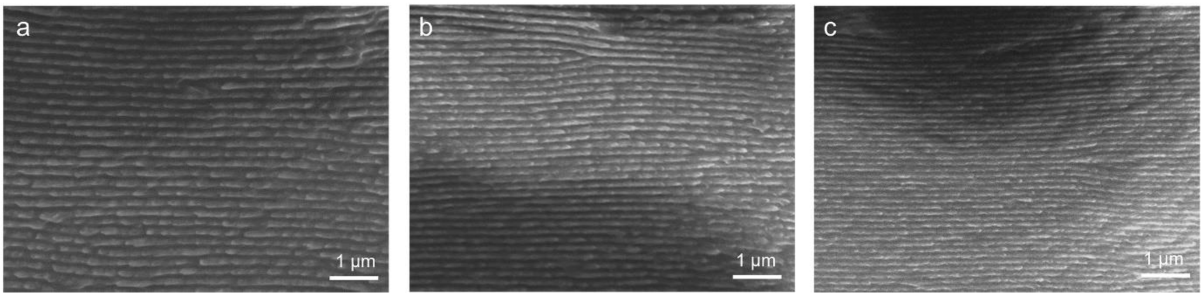

Figure S5. Cross-sectional SEM images of red (a), green (b), and blue (c) HPC-based fibers fabricated from the *ca*. 62 wt%, *ca*. 65 wt% and *ca*. 68 wt% solutions, respectively.

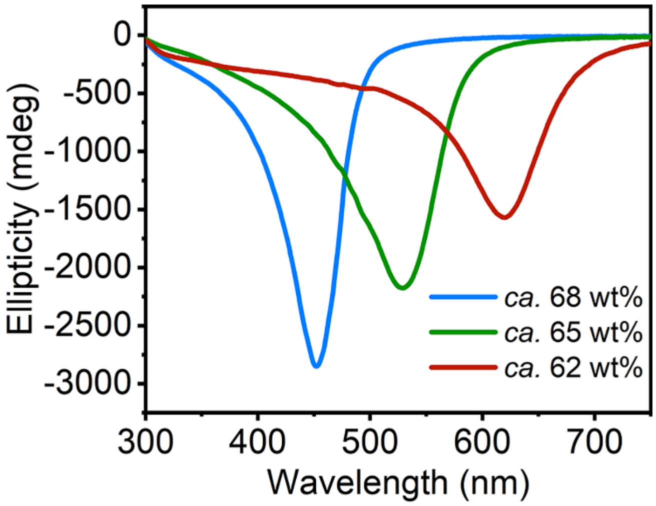

Figure S6. Circular dichroism spectra of the HPC-based fibers fabricated from the *ca*. 62 wt%, *ca*. 65 wt% and *ca*. 68 wt% aqueous solutions, respectively.

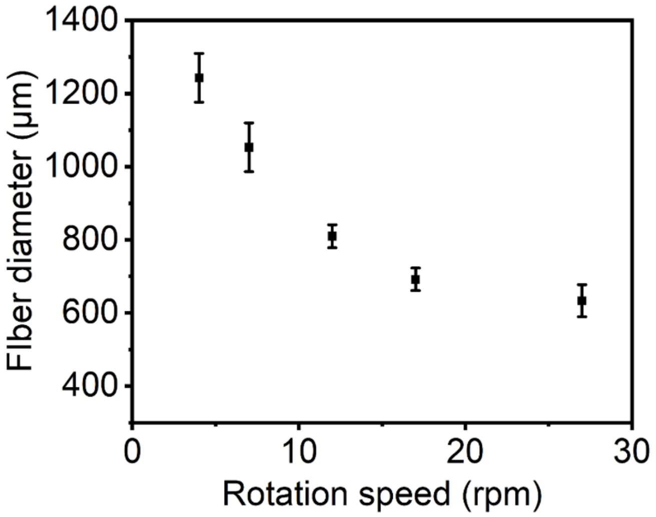

Figure S7. A trend in the fiber diameter as a function of rotation speed of the mandrel. Error bars indicate standard deviation based on 20 measurements.

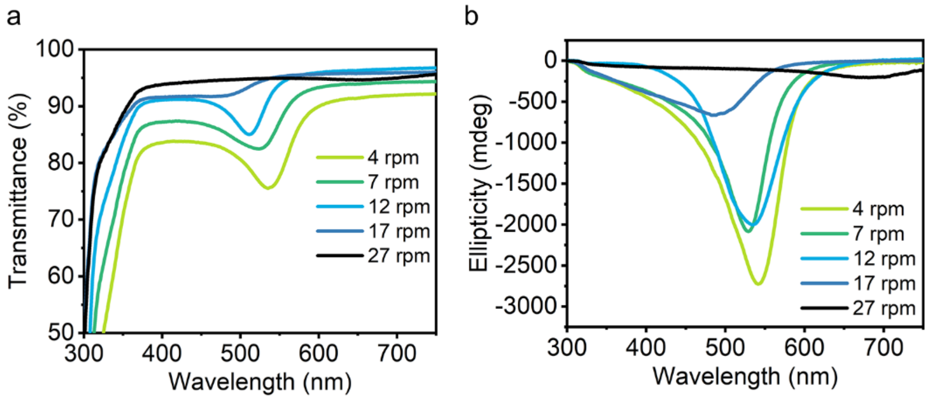

Figure S8. Transmission spectra (a) and CD spectra (b) of the HPC-based fibers fabricated from the *ca*. 65 wt% aqueous solution under different rotation speed of the mandrel.

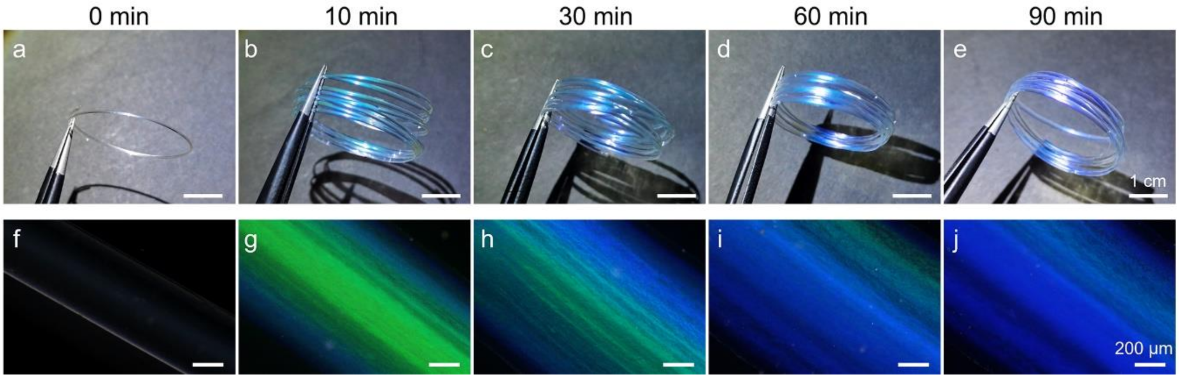

Figure S9. Photographs (a-e) and reflection microscopy images (f-j) of the HPC-based fibers produced with the relaxation time of 0 min (a, f), 10 min (b, g), 30 min (c, h), 60 min (d, i) and 90 min (e, j), respectively.

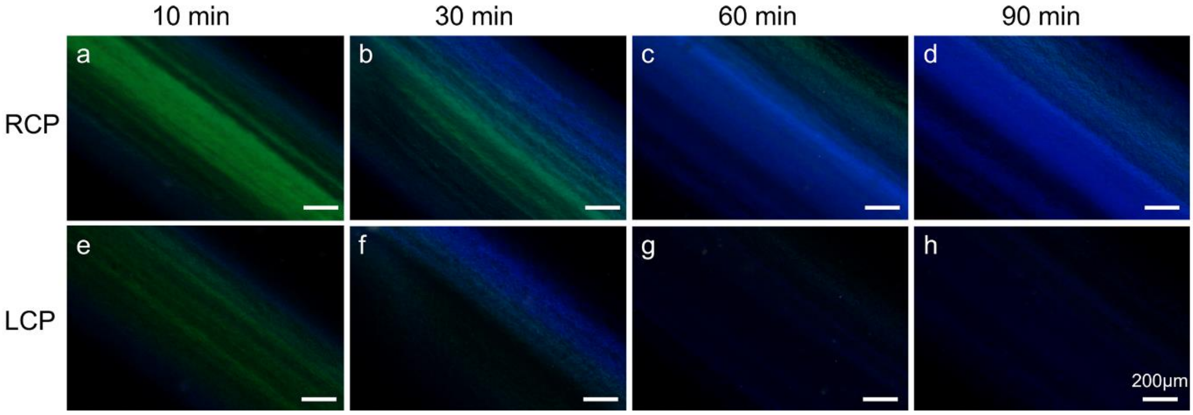

Figure S10. Reflection microscopy images of the HPC-based fibers produced with the relaxation time of 10 min (a, e), 30 min (b, f), 60 min (c, g) and 90 min (d, h) through the RCP filter (a-d) and the LCP filter (e-h).

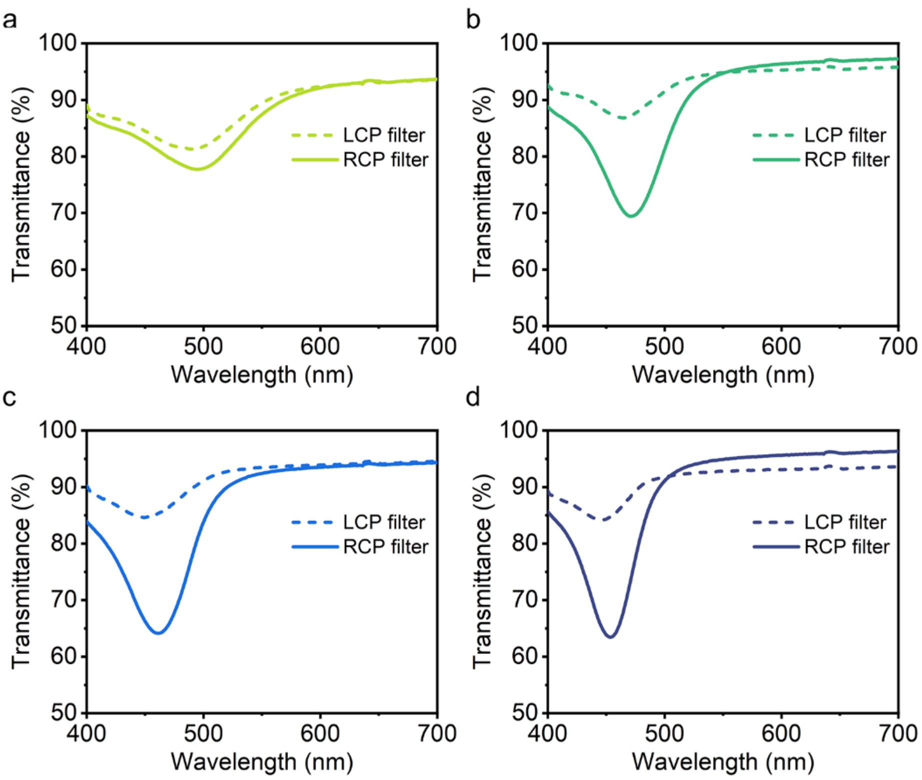

Figure S11. Transmission spectra of the HPC-based fibers produced with the relaxation time of 10 min (a), 30 min (b), 60 min (c), and 90 min (d) recorded through the RCP filter and the LCP filter.

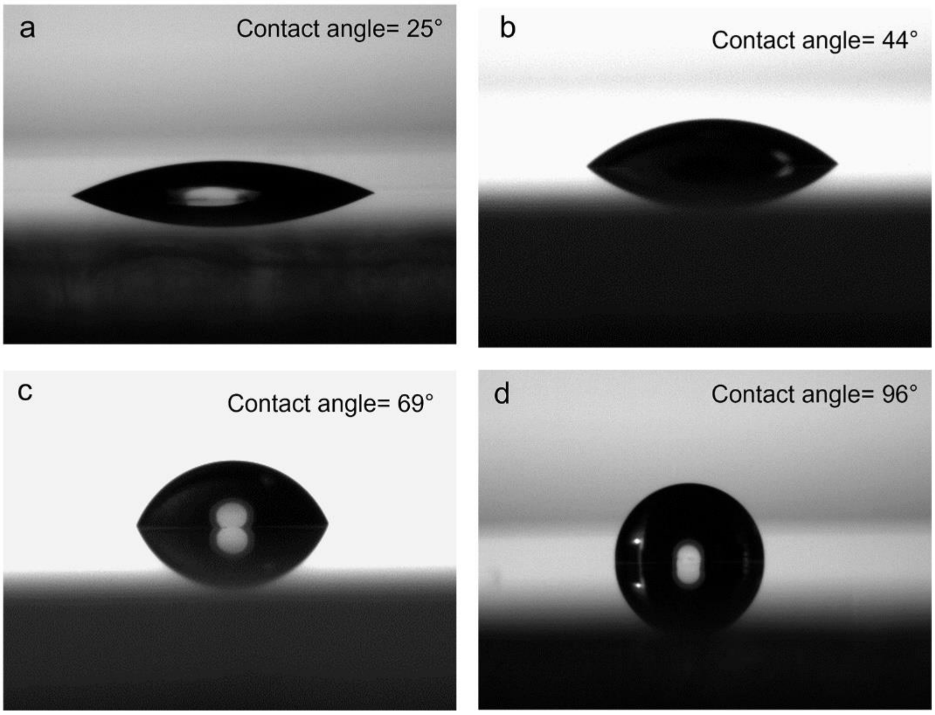

Figure S12. Optical images of the water contact angles on the glass mandrel after different degrees of hydrophobic treatment.

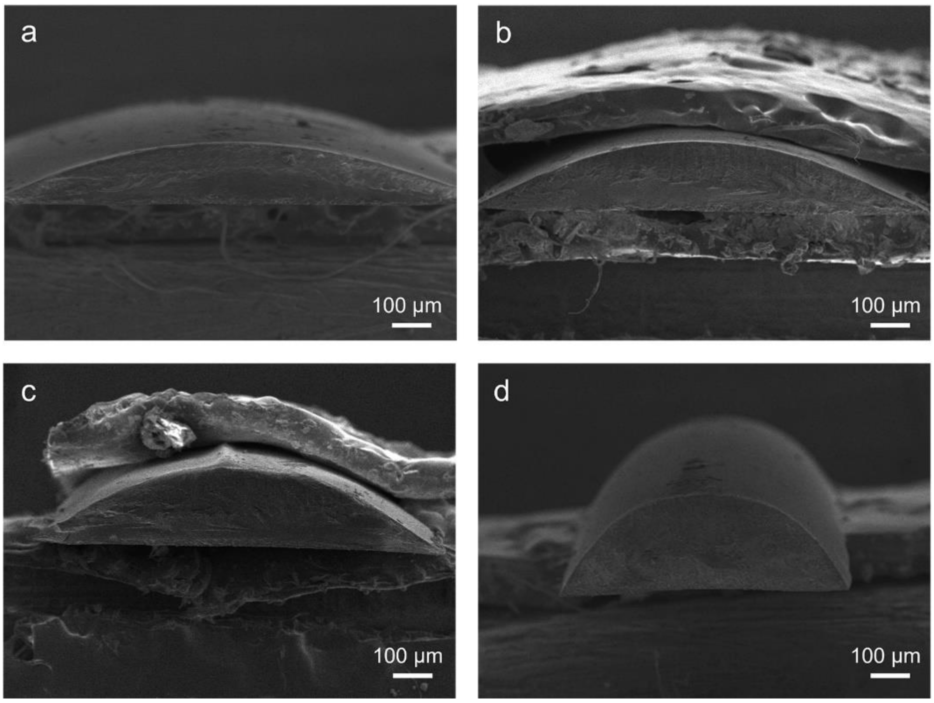

Figure S13. Cross-sectional SEM images of the HPC-based fibers collected on the glass mandrel with the contact angles of 25° (a), 44° (b), 69° (c) and 96° (d), respectively.

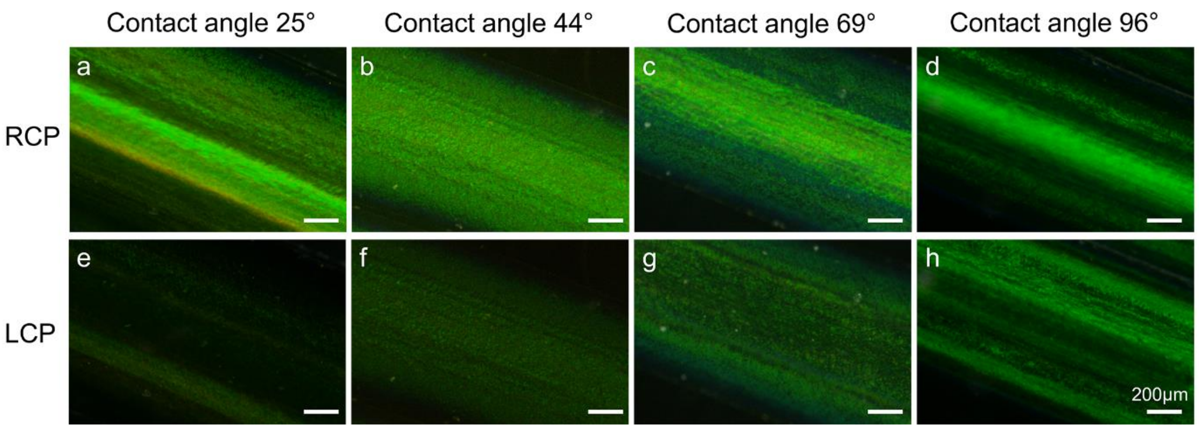

Figure S14. Reflection microscopy images of the HPC-based fibers collected on the glass mandrel with the contact angles of 25° (a, e), 44° (b, f), 69° (c, g) and 96° (d, h) through the RCP filter (a-d) and the LCP filter (e-h).

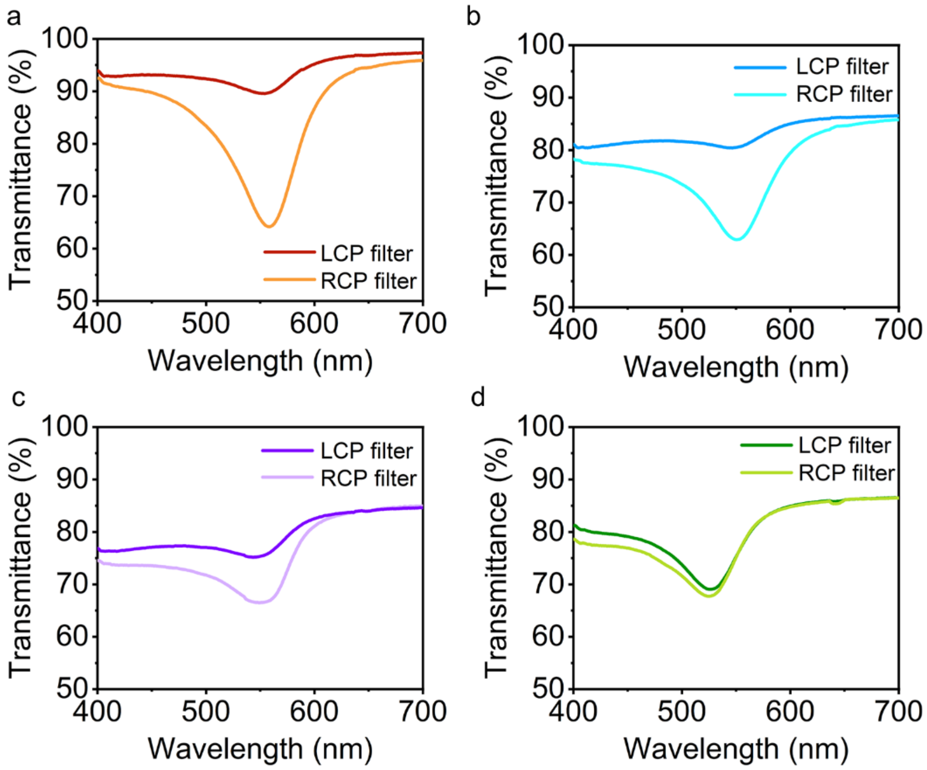

Figure S15. The transmission spectra of the HPC-based fibers collected on the glass mandrel with the contact angles of 25° (a), 44° (b), 69° (c) and 96° (d) recorded through the LCP filter and the RCP filter, respectively..

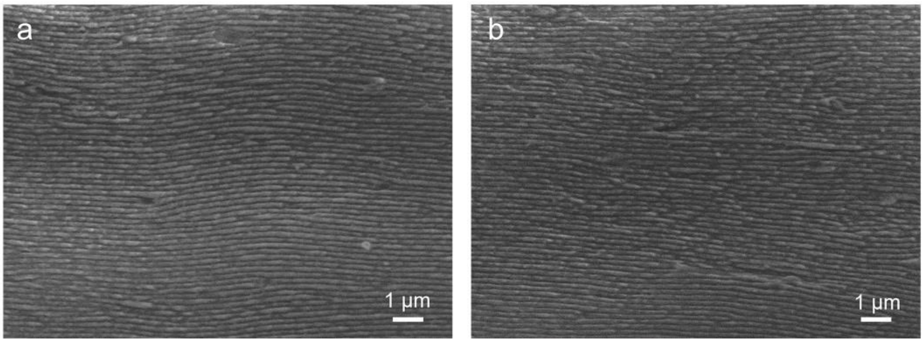

Figure S16. Cross-sectional SEM images of the HPC-based fiber before (a) and after (b) 50 times of bending and twisting treatments.

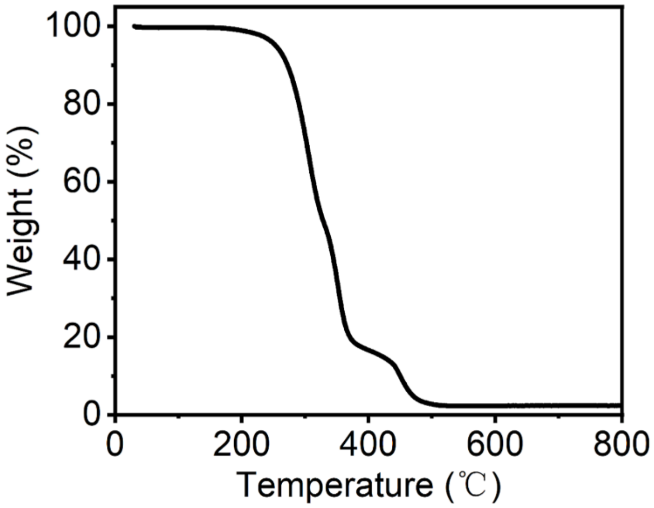

Figure S17. Thermogravimetric analysis (10 °C min^{-1} in air) of the HPC-based fiber.

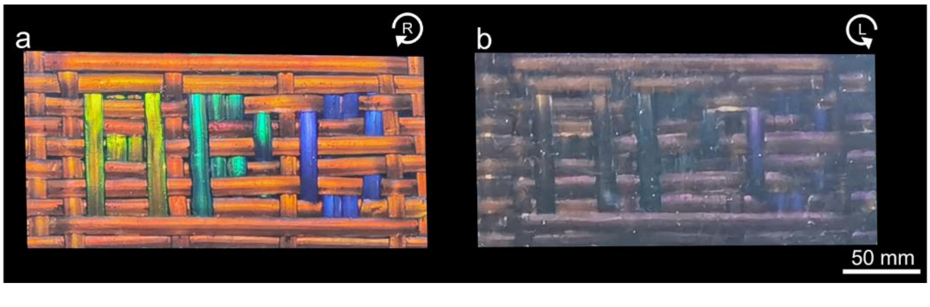

Figure S18. Photographs of a HPC-patterned fabric taken through the RCP filter (a) and the LCP filter (b).

Table S1. Mechanical properties of the HPC-based fibers from the HPC-MA aqueous solutions of different concentrations.

Solution concentration (wt%)	Strength (MPa)	Tensile strain (%)	Young's Modulus (GPa)	Toughness (MJ/cm^3)
62	37.0 ± 3.2	3.2 ± 0.4	1.8 ± 0.2	0.7 ± 0.2
65	39.2 ± 4.4	3.4 ± 0.3	1.9 ± 0.3	0.8 ± 0.1
68	46.4 ± 7.2	4.0 ± 0.6	2.1 ± 0.4	1.2 ± 0.4

Video S1. Production of HPC-based fibers by extruding HPC-MA aqueous solutions using syringe and fiber collection on a rotating glass mandrel.

Video S2. The weight-lifting experiment shows a double stranded HPC-based fiber of 1.20 mm in diameter lifting a 500 g weight.
